# Microsomal Prostaglandin E Synthase-1 Expression in Inflammatory Conditions Is Downregulated by Dexamethasone: Seminal Role of the Regulatory Phosphatase MKP-1

**DOI:** 10.3389/fphar.2017.00646

**Published:** 2017-09-21

**Authors:** Lauri Tuure, Mari Hämäläinen, Brendan J. Whittle, Eeva Moilanen

**Affiliations:** ^1^The Immunopharmacology Research Group, Faculty of Medicine and Life Sciences, University of Tampere, Tampere University Hospital Tampere, Finland; ^2^William Harvey Research Institute, Barts and the London School of Medicine London, United Kingdom

**Keywords:** mPGES-1, prostaglandins, MAP kinases, MKP-1, JNK

## Abstract

Microsomal prostaglandin E synthase-1 (mPGES-1) is an inducible enzyme situated downstream of cyclo-oxygenase-2, promoting the excessive PGE_2_ production in inflammation. Dexamethasone is known to suppress mPGES-1 but the mechanisms regulating mPGES-1 expression remain poorly known. MKP-1 is a phosphatase controlling the proinflammatory MAP kinase pathways p38 and JNK, thus limiting the inflammatory responses. We have now investigated the role of MKP-1 and MAP kinases p38 and JNK in the regulation of mPGES-1 expression by dexamethasone. Dexamethasone increased MKP-1 and decreased mPGES-1 expression in J774 macrophages and in peritoneal macrophages from wild-type but not from MKP-1 deficient mice. Dexamethasone also reduced p38 and JNK phosphorylation along with enhancement of MKP-1, while inhibition of JNK reduced mPGES-1 expression. These findings were also translated to *in vivo* conditions as dexamethasone downregulated mPGES-1 expression in paw inflammation in wild-type but not in MKP-1 deficient mice. In conclusion, dexamethasone was found to downregulate mPGES-1 expression through enhanced MKP-1 expression and reduced JNK phosphorylation in inflammatory conditions. The results extend the understanding on the regulation of mPGES-1 expression and highlight the potential of MKP-1 as an anti-inflammatory drug target.

## Introduction

Microsomal prostaglandin E synthase-1 (mPGES-1) is a terminal enzyme catalyzing the synthesis of prostaglandin E_2_ (PGE_2_), an essential prostanoid linked to pathophysiological conditions, such as inflammation, pain, fever and tumorigenesis (Samuelsson et al., 2007; Stables and Gilroy, 2011; Coulombe et al., 2014; Ruan and So, 2014; Koeberle and Werz, 2015). Two other PGE_2_ synthesizing enzymes have been characterized so far, namely microsomal prostaglandin E synthase-2 (mPGES-2) and cytosolic prostaglandin E synthase (cPGES). Constitutively expressed mPGES-2 and cPGES are presumed to be responsible for physiological PGE_2_ formation, whereas mPGES-1 is an inducible enzyme, the expression of which is induced under inflammatory conditions in various cells and tissues (Samuelsson et al., 2007).

The anti-inflammatory effects of non-steroidal anti-inflammatory drugs (NSAIDs) are considered to be based predominantly on reduced formation of PGE_2_ due to inhibition of cyclo-oxygenase (COX) enzymes. However, the known adverse effects of NSAIDs, such as gastrointestinal, cardiovascular and renal problems are also linked to the same mechanism of action, that is, inhibition of cyclo-oxygenases, restricting the use of NSAIDs among many patient groups. For example, the increased risk of myocardial infarction may in part reflect the reduced formation of prostacyclin (prostaglandin I_2_, PGI_2_) following inhibition of COX-2 in the vascular wall, while mucosal erosions and bleedings in the gastrointestinal track are considered to result from decreased formation of protective physiological prostanoids after inhibition of COX enzymes (Cheng et al., 2006; Grosser et al., 2010; Coxib and traditional, NSAID Trialists' (CNT) Collaboration, et al., 2013).

Such observations could indicate that mPGES-1 is therefore a potential drug target for treating inflammatory conditions and pain by selectively decreasing PGE_2_ production under inflammatory conditions. An inhibitor of mPGES-1 activity or expression could thus lead to a selective decrease of excessive PGE_2_ formation during inflammation since the synthesis of other prostanoids are not reduced similarly, as under the treatment with NSAIDs; furthermore, the constitutive PGE_2_ synthesis through mPGES-2 and cPGES remains unchanged. Moreover, it has been reported that genetic deletion or pharmacological inhibition of mPGES-1 may also lead to an increased production of prostanoids exhibiting anti-inflammatory properties (Idborg et al., 2013) and possibly modulate platelet function during inflammation (Raouf et al., 2016). Overall, inhibition of mPGES-1 may therefore result in therapeutic activity comparable to NSAIDs but with less adverse effects (Samuelsson et al., 2007; Korotkova and Jakobsson, 2013; Koeberle and Werz, 2015).

Inhibitors of mPGES-1 activity are indeed under development. Recently, a phase I trial with the selective mPGES-1 inhibitor LY3023703 indicated a decreased level of a urinary metabolite of PGE_2_ to an extent comparable to that caused by the COX-2 inhibitor celecoxib. In addition, a more favorable effect on the production of prostanoids other than PGE_2_ was found (Jin et al., 2015). Development of mPGES-1 inhibitors currently appears to be focused on molecules affecting the activity of mPGES-1 and subsequent PGE_2_ production (Chang and Meuillet, 2011; Korotkova and Jakobsson, 2013; Koeberle and Werz, 2015; Chandrasekhar et al., 2016; Gupta and Aparoy, 2016; Koeberle et al., 2016). However, as presented in this study, an alternative approach might be to focus on the downregulation of the expression of mPGES-1 rather than directly affecting the activity of the enzyme.

Mitogen activated protein (MAP) kinases, namely extracellular signal-regulated kinase (ERK), p38 MAP-kinase and c-Jun N-terminal kinase (JNK), regulate the cellular response to various extracellular inflammatory stimuli (Johnson and Lapadat, 2002; Raman et al., 2007). These enzymes are activated by phosphorylation and play an essential role in the promotion of inflammatory responses and innate immune system (Kramer et al., 1996; Ashwell, 2006; Rincon and Davis, 2009; Plotnikov et al., 2011; Korhonen and Moilanen, 2014). Mitogen-activated protein kinase phosphatases (MKPs) inactivate MAP kinases through dephosphorylation and form a negative feedback system for the activity of MAP kinases, thus controlling and limiting the inflammatory reaction and innate immune responses (Chi et al., 2006; Hammer et al., 2006; Zhao et al., 2006; Wang and Liu, 2007; Li et al., 2009; Wancket et al., 2012). MKP-1 is the most studied of MKP enzymes and it has been shown to dephosphorylate MAP kinases p38 and / or JNK, depending on the cell type, and thereby decreasing the expression of many MAP kinase-dependent pro-inflammatory factors (Franklin and Kraft, 1997; Franklin et al., 1998; Chi et al., 2006; Zhao et al., 2006; Turpeinen et al., 2010, 2011; Comalada et al., 2012). MKP-1 has been identified as a mediator of some anti-inflammatory effects of drugs such as the glucocorticoids, the anti-rheumatic drug aurothiomalate, phosphodiesterase 4 inhibitors (Kassel et al., 2001; Abraham et al., 2006; Nieminen et al., 2010; Shipp et al., 2010; Korhonen et al., 2013; Keränen et al., 2017) and recently, also β_2_-agonists (Keränen et al., 2016). In addition, inflammatory responses have been found to be more severe and even lethal in MKP-1 knock-out mice as compared to wild-type controls (Chi et al., 2006; Zhao et al., 2006; Frazier et al., 2009; Korhonen et al., 2011, 2013), emphasizing the role of MKP-1 as a limiting factor in inflammation.

Mechanisms regulating the expression of mPGES-1 are not fully known, but one of the few drugs found to inhibit the expression of mPGES-1 is the glucocorticoid dexamethasone (Stichtenoth et al., 2001), known also to enhance the expression of MKP-1 (Kassel et al., 2001; Abraham et al., 2006; Shipp et al., 2010). Therefore, we tested the hypothesis that dexamethasone inhibits the expression of mPGES-1 via an elevated MKP-1 expression and subsequent dephosphorylation of MAP kinases, by using the J774 macrophage cell line and confirmed the findings by using peritoneal macrophages from MKP-1 deficient and corresponding wild-type mice. To evaluate the significance of these findings *in vivo*, the effects of dexamethasone on the expression of mPGES-1 in mouse paw inflammation in MKP-1 deficient and corresponding wild-type mice was also studied.

## Materials and methods

### Materials

Dexamethasone was received from Orion Corp. (Espoo, Finland). Lipopolysaccharide (LPS) from Escherichia coli strain 0111:B4 and all other reagents were purchased from Sigma-Aldrich Inc. (St. Louis, MO, USA) unless otherwise stated.

### Animals

Wild-type and MKP-1(-/-) C57BL/6 mice originally generated in the laboratory of R. Bravo at Bristol-Myers Squibb Pharmaceutical Research Institute (Princeton, NJ, USA) were used in the present study. Mice were bred at the animal facilites in Faculty of Medicine and Life Sciences, University of Tampere under standard conditions (12:12 light-dark cycle, +22 ± 1°C temperature, 50–60% humidity), and food and water provided *ad libitum*. Animal experiments were carried out in accordance with the legislation for the protection of animals used for scientific purposes (Directive 2010/63/EU), and the study was approved by the National Animal Experiment Board.

### Lipopolysaccharide-induced paw edema

Lipopolysaccharide [50 μl of 2 mg/ml in phosphate-buffered saline (PBS)] was injected into the hind paw of anesthetized mice (0.5 mg/kg, Domitor®; Orion Oyj, Espoo, Finland, and 75 mg/kg, Ketalar®; Pfizer Oy Animal Health, Helsinki, Finland). The contralateral paw was injected with the corresponding volume of endotoxin-free PBS. Mice were treated 1 h prior to the injection of LPS with dexamethasone (2 mg/kg intraperitoneally) or with vehicle (PBS). Paw volumes were measured up to 6 h with a plethysmometer (Ugo Basile, Comerio, Italy) and compared to the baseline value. After the last measurement mice were sacrified (cervical dislocation) and paw tissues were collected into RNA Later solution (Invitrogen, Carlsbad, CA, USA).

### Cell culture

J774 mouse macrophages (American Type Culture Collection, Rockville Pike, MD, USA) were cultured at +37°C in 5% CO_2_ atmosphere in Dulbecco's Modified Eagle's medium (DMEM, Invitrogen, Paisley, UK) containing 10% (v/v) heat-inactivated fetal bovine serum (FBS), 100 U/ml penicillin, 100 μg/ml streptomycin and 250 ng/ml amphotericin B (all from Gibco, Wien, Austria). Cells (2.5 × 10^5^ per well) were seeded on 24-well plates and the cell monolayers were grown for 72 h prior to the experiments. SP600125 and SB203580 were dissolved in dimethyl sulfoxide (DMSO), dexamethasone and LPS in PBS. LPS and the compounds under investigation in concentrations indicated or the solvent (DMSO, final concentration 0.1% v/v in all wells) were added to the cells in fresh culture medium containing 10% FBS and the supplements, and the incubations were continued for the time indicated before removing the culture medium and harvesting the cells.

Mouse peritoneal macrophages were obtained by peritoneal lavage with sterile PBS supplemented with 0.2 mM ethylenediaminetetraacetic acid (EDTA). Cells were washed and seeded on 24-well plates (1 × 10^6^ cells/well) in RPMI medium supplemented with 2% FBS, 100 U/ml penicillin, 100 μg/ml streptomycin and 250 ng/ml amphotericin B. Cells were incubated overnight, washed and treated with the compounds under investigation for the period indicated.

### Preparation of cell lysates and western blot analysis

At the indicated time points, the culture medium was removed and the cells were washed with ice-cold PBS and solubilized in cold lysis buffer containing 10 mM Tris–HCl, 5 mM EDTA, 50 mM NaCl, 1% Triton X-100, 0.5 mM phenylmethylsulfonyl fluoride, 1 mM sodium orthovanadate, 20 mg/ml leupeptin, 50 mg/ml aprotinin, 5 mM sodium fluoride, 2 mM sodium pyrophosphate and 10 mM *n*-octyl-b-D-glucopyranoside. After incubation for 15 min on ice, lysates were centrifuged, and the supernatants were collected and mixed in a ratio of 1:4 with SDS loading buffer (62.5 mM Tris–HCl, pH 6.8, 10% glycerol, 2% SDS, 0.025% bromophenol blue and 5% β-mercaptoethanol), and stored at −20°C until analyzed.

Equal amounts of protein (10 or 20 μg) were loaded on a 12% SDS-polyacrylamide gel and separated by electrophoresis. Proteins were transferred to nitrocellulose membranes by dry electroblotting using iBlot gel transfer stacks and the Invitrogen iBlot Device according to the manufacturer's instructions. After transfer, the membrane was blocked in TBS/T [20 mM Trisbase (pH 7.6), 150 mM NaCl, 0.1% Tween-20] containing 5% non-fat milk for 1 h at room temperature. For detection of phosphorylated proteins, membranes were blocked in TBS/T containing 5% BSA. Membranes were incubated overnight at 4°C with the primary antibody and for 1 h with the secondary antibody, and the chemiluminescent signal was detected by ImageQuant™ LAS 4000 mini (GE Healthcare Bio-Sciences AB, Uppsala, Sweden). The chemiluminescent signal was quantified with ImageQuant TL 7.0 Image Analysis Software (GE Healthcare Bio-Sciences AB). Following antibodies were used in the Western blot analysis: mPGES-1 antibody (AS-03031; Agrisera AB, Vännäs, Sweden); polyclonal goat anti-rabbit (sc-2004), actin (sc-1616R) and JNK antibody (#9251; Santa Cruz Biotechnology, CA, USA), MKP-1 antibody (SAB2500331; Sigma-Aldrich Inc), p38 MAPK antibody (ab27986; Abcam plc., Cambridge, UK), phospho-p38 MAPK (#9211) and phospho-JNK antibody (#9251; Cell Signaling Technology Inc., Beverly, MA, USA).

### RNA extraction and quantitative real-time reverse transcription polymerase chain reaction (qRT-PCR)

At the indicated time points, the culture medium was removed, and cell homogenization and RNA extraction was carried out by using GenElute™ Mammalian Total RNA Miniprep Kit according to the manufacturer's instruction. In the case of paw tissue samples, RNA was extracted with TRIzol reagent. Briefly, tissue was first homogenized in TRIzol (Thermo Fisher Scientific, Waltham, MA, USA), and thereafter RNA was extracted with chloroform and precipitated with isopropanol, washed with 75% ethanol and resuspended in RNAse free water. Reverse transcription of the RNA to cDNA was performed with TaqMan® Reverse Transcription Reagents (Applied Biosystems, Foster City, CA, USA) in the case of J774 cells and with Maxima First strand cDNA synthesis kit for RT-qPCR (Thermo Fisher Scientific) in the case of PM cells and paw tissue.

Primers and probes were purchased from Metabion (Martinsried, Germany). Their sequences and concentrations were optimized according to the manufacturer's guidelines in TaqMan Universal PCR Master Mix Protocol part number 4304449 revision C (Applied Biosystems) and were as follows: mouse mPGES-1 CCTGGATACATTTCCTCGTTGTC (forward, 300 nM), GAAGGCGTGGGTTCAGCTT (reverse, 300 nM), and ACAGGCCGTGTGGTACACACCG (probe, 150 nM); mouse MKP-1 CTCCTGGTTCAACGAGGCTATT (forward, 300 nM), TGCCGGCCTGGCAAT (reverse, 300 nM), and CCATCAAGGATGCTGGAGGGAGAGTGTT (probe, 150 nM); mouse GAPDH GCATGGCCTTCCGTGTTC (forward, 300 nM), GATGTCATCATACTTGGCAGGTTT (reverse, 300 nM) and TCGTGGATCTGACGTGCCGCC (probe, 150 nM). Quantitative PCR was carried out by using TaqMan Universal PCR Master Mix and ABI Prism 7500 sequence detection system (Applied Biosystems). The PCR cycling parameters were incubation at 50°C for 2 min, incubation at 95°C for 10 min, 40 cycles of denaturation at 95°C for 15 s and annealing and extension at 60°C for 1 min. A standard curve method was used to estimate the relative mRNA levels. When calculating the results, mPGES-1 and MKP-1 mRNA levels were first normalized against GAPDH.

### Statistics

Results are expressed as mean + standard error of the mean (SEM). One-way ANOVA with Bonferroni's post-test was performed using GraphPad InStat version 3.10 for Windows. Differences were considered significant at ^*^P < 0.05, ^**^P < 0.01 or ^***^P < 0.001.

## Results

**Figure 1:**
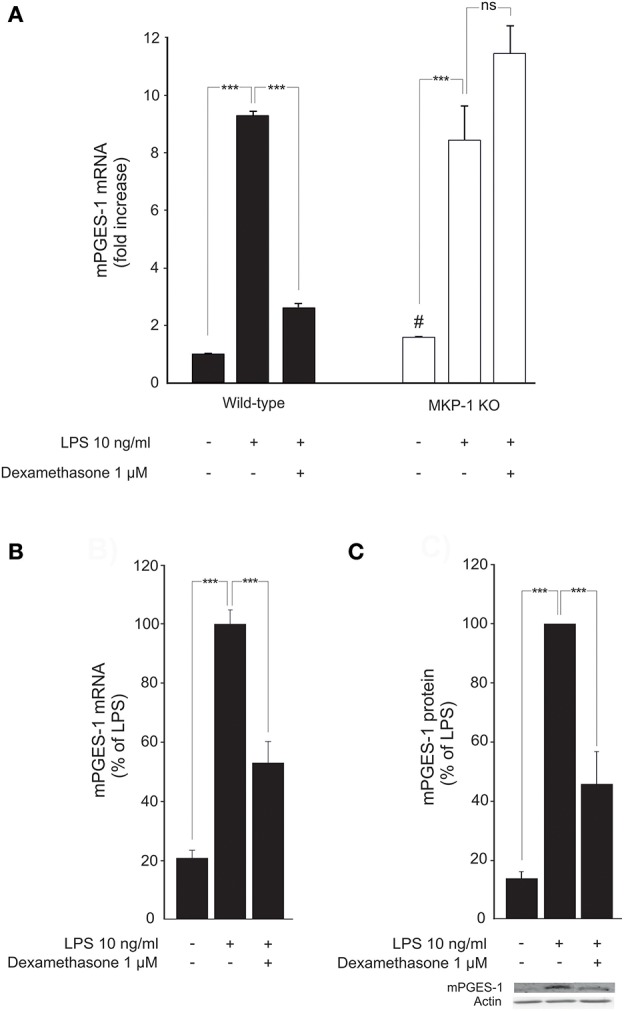
Dexamethasone inhibits mPGES-1 expression in activated macrophages in an MKP-1 dependent manner. **(A)** Effects of dexamethasone on peritoneal macrophages from wild-type and MKP-1 knock-out (KO) mice. Cells were incubated with LPS in the presence or absence of dexamethasone for 24 h. mPGES-1 mRNA levels were measured by quantitative RT-PCR and normalized against GAPDH mRNA levels. Results are expressed in arbitrary units, mPGES-1 mRNA levels in unstimulated cells from wild type mice were set as 1, and the other values were related to that. Results are expressed as mean + SEM, *n* = 4. One-way ANOVA with Bonferroni's post-test was performed and statistical significance is indicated as ^***^P < 0.001 and ns = not significant. ^#^*P* = 0.0286 vs unstimulated cells from wild-type mice. **(B)** Effect of dexamethasone on mPGES-1 mRNA production in J774 murine macrophages. Cells were stimulated with LPS in the presence or absence of dexamethasone for 24 h. mPGES-1 mRNA levels were measured by quantitative RT-PCR and normalized against GAPDH mRNA levels. Results are expressed in arbitrary units, mPGES-1 mRNA levels in LPS-stimulated cells were set as 100 % and the other values were related to that. Results are expressed as mean + SEM, *n* = 6–7. One-way ANOVA with Bonferroni's post-test was performed and statistical significance is indicated as ^***^P < 0.001. **(C)** Effect of dexamethasone on mPGES-1 protein expression in J774 murine macrophages. Cells were stimulated with LPS in the presence or absence of dexamethasone for 24 h. mPGES-1 protein levels were measured by Western blot analysis and actin was used as a loading control. Results are expressed in arbitrary units, mPGES-1 protein levels in LPS-stimulated cells were set as 100% and the other values were related to that. Results are expressed as mean + SEM, *n* = 6. One-way ANOVA with Bonferroni's post-test was performed and statistical significance is indicated as ^***^P < 0.001. Shown is a representative gel of six with similar results.

Expression of mPGES-1 in unstimulated macrophages from MKP-1 deficient mice was elevated as compared to macrophages from wild-type mice (Figure 1A). A noticeable increase in the expression level was seen following stimulation with LPS both in mouse peritoneal macrophages (Figure 1A) and in J774 macrophage cell line (Figures 1B,C). Incubation with dexamethasone significantly downregulated mPGES-1 expression in both LPS-stimulated J774 macrophages (Figures 1B,C) and in peritoneal macrophages from wild-type mice (Figure 1A).

In contrast, incubation with dexamethasone had no effect on LPS-induced mPGES-1 expression in peritoneal macrophages from MKP-1 deficient mice (Figure 1A). This suggests that MKP-1 has an essential role in mediating the suppression by dexamethasone of mPGES-1 expression in inflammation.

**Figure 2:**
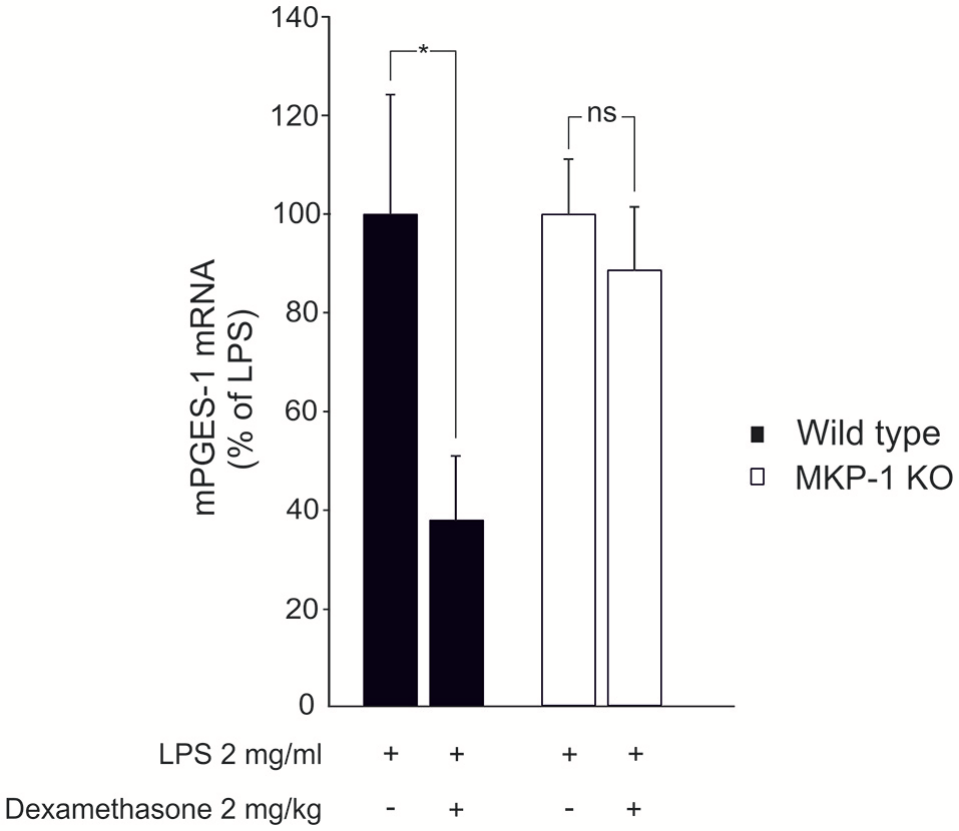
Dexamethasone inhibits mPGES-1 expression in acute inflammatory response *in vivo* in an MKP-1 dependent manner. Dexamethasone (2 mg/kg) was given intraperitoneally an hour before LPS (50 μl of 2 mg/ml in PBS) was injected into the hind paw of anesthetized mice to induce acute inflammation. The paw tissues were collected 6 h after the LPS injection and mPGES-1 mRNA levels were measured by quantitative RT-PCR and normalized against GAPDH mRNA levels. Results are expressed in arbitrary units, mPGES-1 mRNA levels in LPS treated paw tissue from wild-type mice were set as 100% and the other values are related to that. Results are expressed as mean + SEM, *n* = 6–8. One-way ANOVA with Bonferroni's post-test was performed and statistical significance is indicated as ^*^P < 0.05 and ns = not significant.

**Figure 3:**
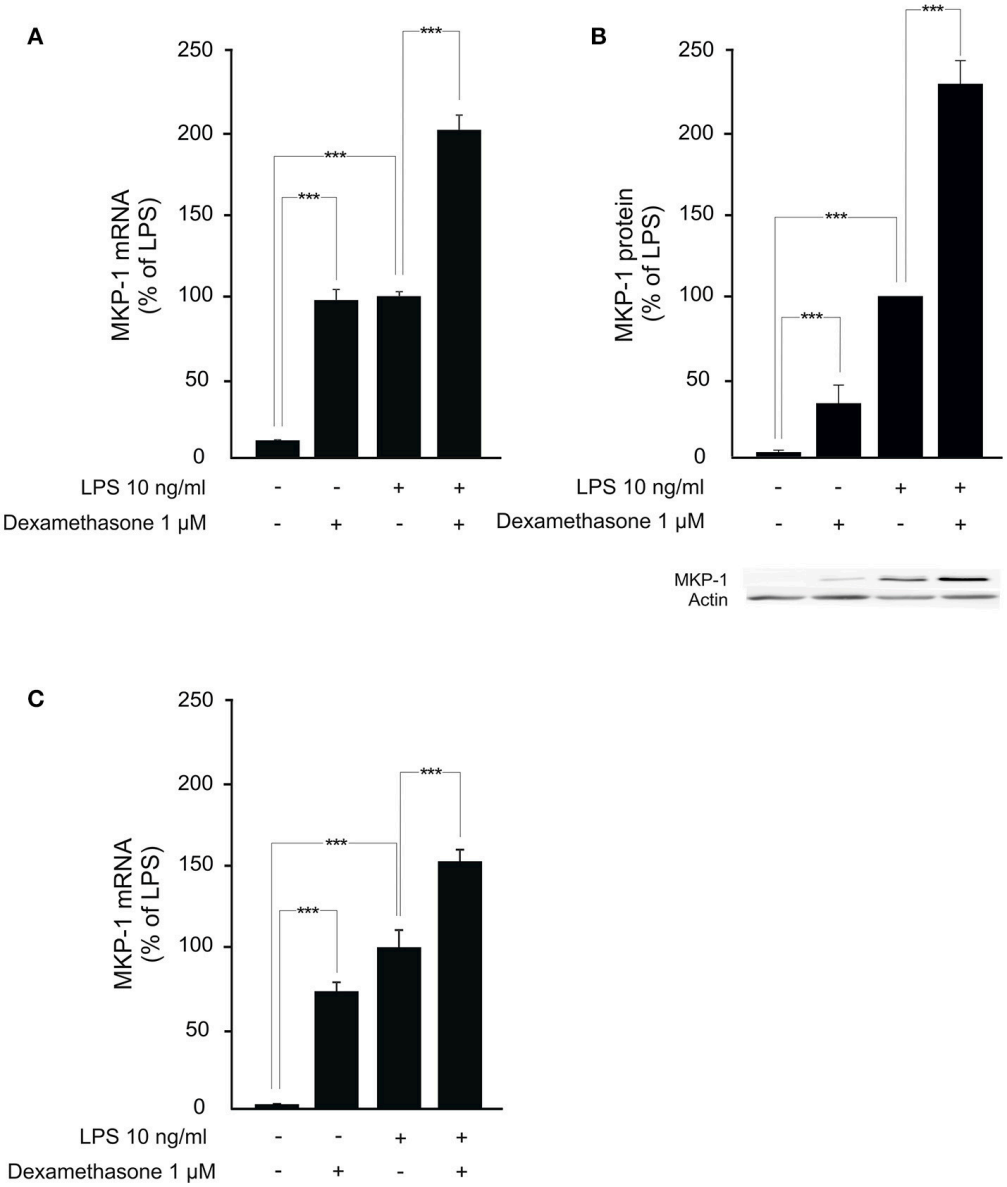
Dexamethasone enhances MKP-1 expression in macrophages. **(A)** Effect of dexamethasone on MKP-1 mRNA expression in J774 murine macrophages. Cells were stimulated with LPS in the presence or absence of dexamethasone for 1 h. MKP-1 mRNA levels were measured by quantitative RT-PCR and normalized against GAPDH mRNA levels. Results are given in arbitrary units, MKP-1 mRNA levels in LPS-stimulated cells were set as 100% and the other values were related to that. Results are expressed as mean + SEM, *n* = 11–12. One-way ANOVA with Bonferroni's post-test was performed and statistical significance is indicated as ^***^P < 0.001. **(B)** Effect of dexamethasone on MKP-1 protein expression in J774 murine macrophages. Cells were stimulated with LPS in the presence or absence of dexamethasone for 1 h. MKP-1 protein levels were measured by Western blot analysis and actin was used as a loading control. Results are expressed in arbitrary units, MKP-1 protein levels in LPS-stimulated cells were set as 100% and the other values were related to that. Results are expressed as mean + SEM. *n* = 9. One-way ANOVA with Bonferroni's post-test was performed and statistical significance is indicated as ^***^P < 0.001. Shown is a representative gel of nine with similar results. **(C)** Effect of dexamethasone on MKP-1 mRNA production in peritoneal macrophages from wild-type mice. Cells were stimulated with LPS in the presence or absence of dexamethasone for 1 h. MKP-1 mRNA levels were measured by quantitative RT-PCR and normalized against GAPDH mRNA levels. Results are expressed in arbitrary units, MKP-1 mRNA levels in LPS-stimulated cells were set as 100% and the other values were related to that. Results are expressed as mean + SEM, *n* = 5. One-way ANOVA with Bonferroni's post-test was performed and statistical significance is indicated as ^***^P < 0.001.

We next investigated whether MKP-1 could mediate the attenuation by dexamethasone of mPGES-1 expression also under *in vivo* conditions. To do this, the effect of dexamethasone treatment on mPGES-1 expression in paw inflammation in wild-type and MKP-1 deficient mice was examined. Dexamethasone, in a dose (2 mg/kg intraperitoneally) that inhibited the concurrent paw edema (by 44%; *P* < 0.05) in wild-type but not in MKP-1 deficient mice, significantly reduced mPGES-1 expression in LPS-treated paw tissue in wild-type mice. In support of the *in vitro* data, dexamethasone had no effect on mPGES-1 expression levels in the paw tissue in MKP-1 deficient mice (Figure 2). To confirm that dexamethasone could stimulate MKP-1 expression in macrophages, MKP-1 mRNA and protein levels in J774 cells were measured. MKP-1 expression was low in unstimulated cells but it was increased by LPS. Furthermore, dexamethasone enhanced MKP-1 mRNA (Figure 3A) and protein (Figure 3B) levels in J774 macrophages both in the absence and in the presence of LPS. Dexamethasone likewise enhanced MKP-1 expression in unstimulated and LPS-stimulated peritoneal macrophages from wild-type mice (Figure 3C).

**Figure 4:**
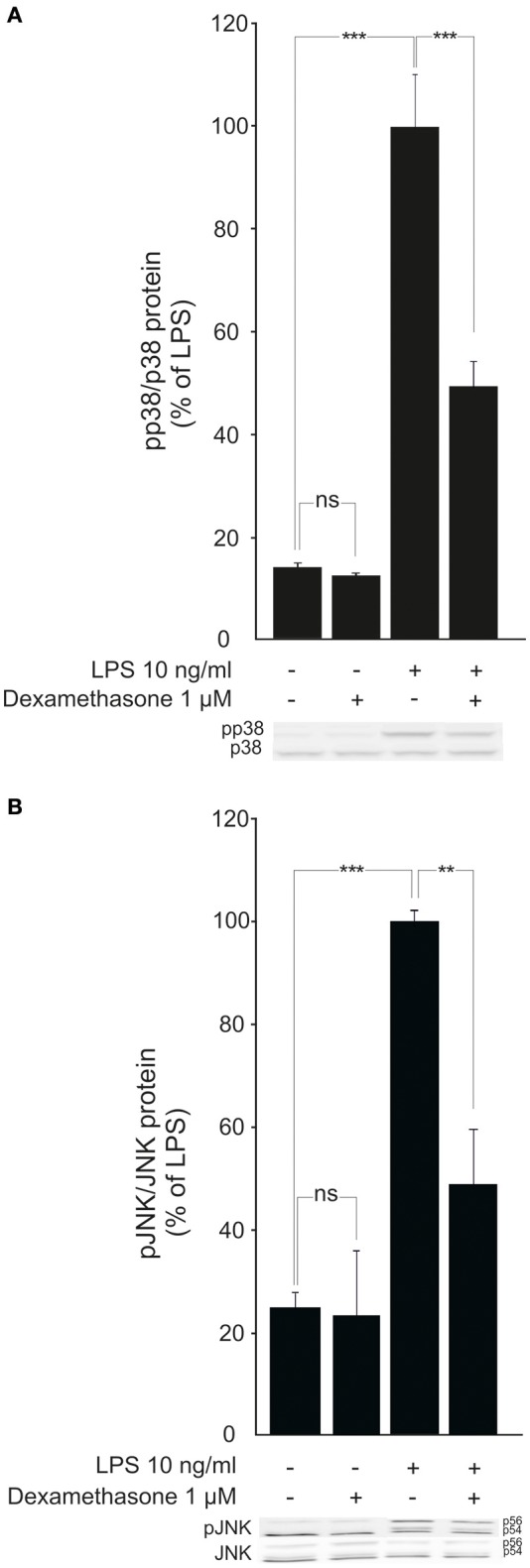
Dexamethasone inhibits the phosphorylation of MAP kinases p38 and JNK in activated J774 macrophages. **(A)** Effect of dexamethasone on p38 phosphorylation. J774 macrophages were preincubated with dexamethasone for 1 h and stimulated with LPS for 30 min. p38 and phosphorylated p38 (pp38) protein levels were measured by Western blot analysis and the levels of phosphorylated p38 were normalized against the levels of total p38. Results are expressed in arbitrary units, phosphorylated p38 levels in LPS-stimulated cells were set as 100% and the other values were related to that. Results are expressed as mean + SEM, *n* = 4. One-way ANOVA with Bonferroni's post-test was performed and statistical significance is indicated as ^***^P < 0.001 and ns = not significant. Shown is a representative gel of four with similar results. **(B)** Effect of dexamethasone on JNK phosphorylation. J774 macrophages were preincubated with dexamethasone for 1 h and stimulated with LPS for 30 min. JNK and phosphorylated JNK (pJNK) protein levels were measured by Western blot analysis and the levels of phosphorylated JNK were normalized against the levels of total JNK. Results are expressed in arbitrary units, phosphorylated JNK levels in LPS-stimulated cells were set as 100% and the other values were related to that. Results are expressed as mean + SEM, *n* = 6. One-way ANOVA with Bonferroni's post-test was performed and statistical significance is indicated as ^**^P < 0.01, ^***^P < 0.001 and ns = not significant. Shown is a representative gel of six with similar results.

**Figure 5:**
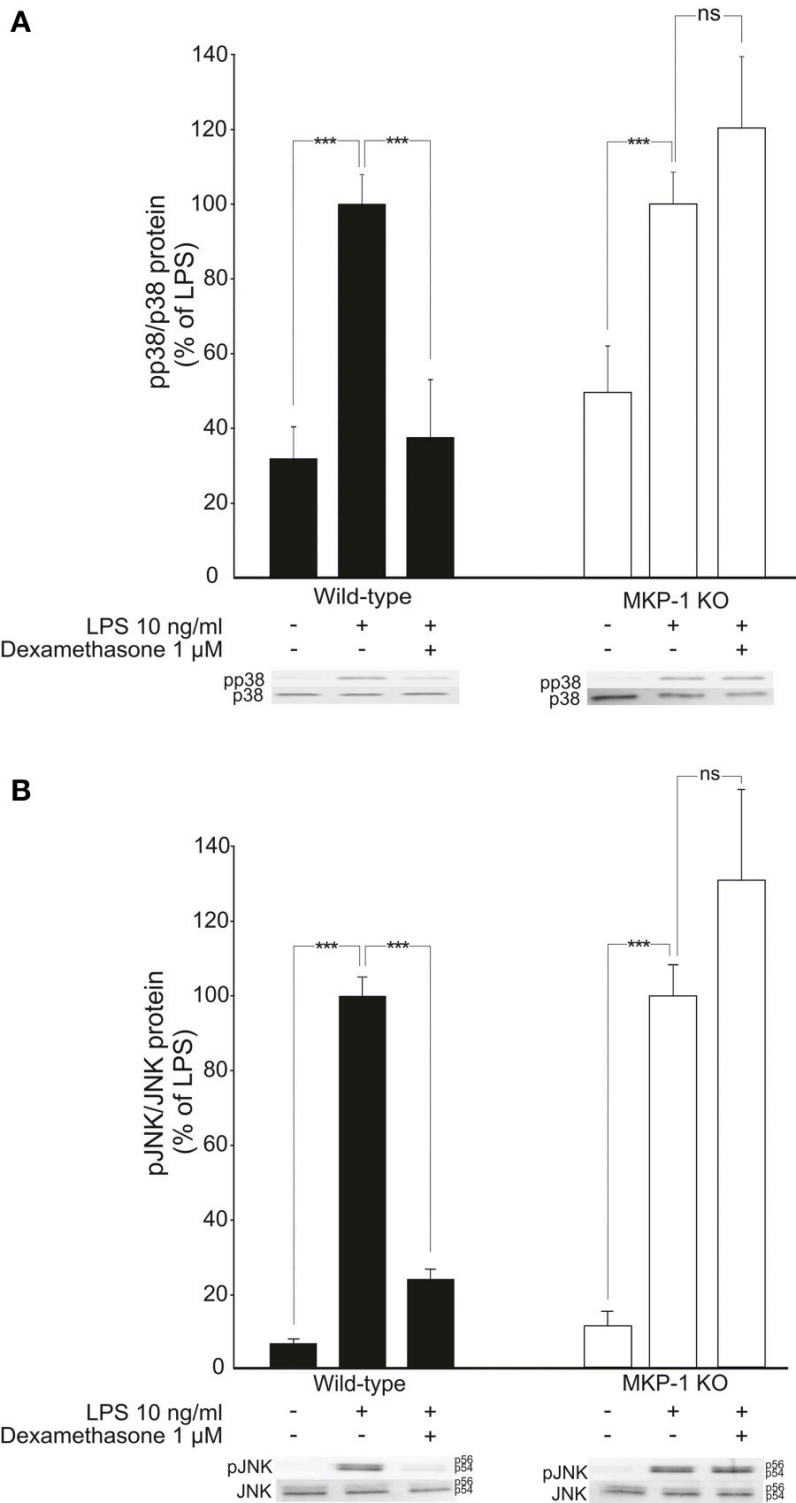
Dexamethasone inhibits the phosphorylation of MAP kinases p38 and JNK in activated macrophages in an MKP-1 dependent manner. **(A)** Effect of dexamethasone on p38 phosphorylation. Peritoneal macrophages from wild-type and MKP-1 knock-out (KO) mice were preincubated with dexamethasone for 1 h and stimulated with LPS for 30 min. p38 and phosphorylated p38 (pp38) protein levels were measured by Western blot and the levels of phosphorylated p38 were normalized against the levels of total p38. Results are expressed in arbitrary units, phosphorylated p38 levels in LPS-stimulated cells were set as 100 % and the other values were related to that. Results are expressed as mean + SEM, *n* = 9. One-way ANOVA with Bonferroni's post-test was performed and statistical significance is indicated as ^***^P < 0.001 and ns = not significant. Shown is a representative gel of nine with similar results. **(B)** Effect of dexamethasone on JNK phosphorylation. Peritoneal macrophages from wild-type and MKP-1 knock-out (KO) mice were pre-incubated with dexamethasone for 1 h and stimulated with LPS for 30 min. JNK and phosphorylated JNK (pJNK) protein levels were measured by Western blot analysis and the levels of phosphorylated JNK were normalized against the levels of total JNK. Results are expressed in arbitrary units, phosphorylated JNK levels in LPS-stimulated cells were set as 100% and the other values were related to that. Results are expressed as mean + SEM, *n* = 5. One-way ANOVA with Bonferroni's post-test was performed and statistical significance is indicated as ^***^P < 0.001 and ns = not significant. Shown is a representative gel of five with similar results.

Because MKP-1 has been reported to inactivate p38 and JNK MAP kinases through dephosphorylation (Franklin and Kraft, 1997; Franklin et al., 1998; Kassel et al., 2001; Abraham et al., 2006; Chi et al., 2006; Zhao et al., 2006; Turpeinen et al., 2010, 2011; Comalada et al., 2012), the effects of dexamethasone on the levels of phosphorylated p38 and JNK in activated macrophages were investigated. Exposure to LPS caused a rapid increase in p38 and JNK phosphorylation in J774 macrophages (Figures 4A,B) and in mouse peritoneal macrophages (Figures 5A,B). This effect was reduced by dexamethasone in J774 cells (Figures 4A,B) and in peritoneal macrophages from wild-type mice (Figures 5A,B). The role of MKP-1 in the dexamethasone effect was confirmed by the finding that dexamethasone did not reduce the LPS-enhanced levels of phosphorylated p38 (Figure 5A) or JNK (Figure 5B) in peritoneal macrophages from MKP-1 deficient mice.

**Figure 6:**
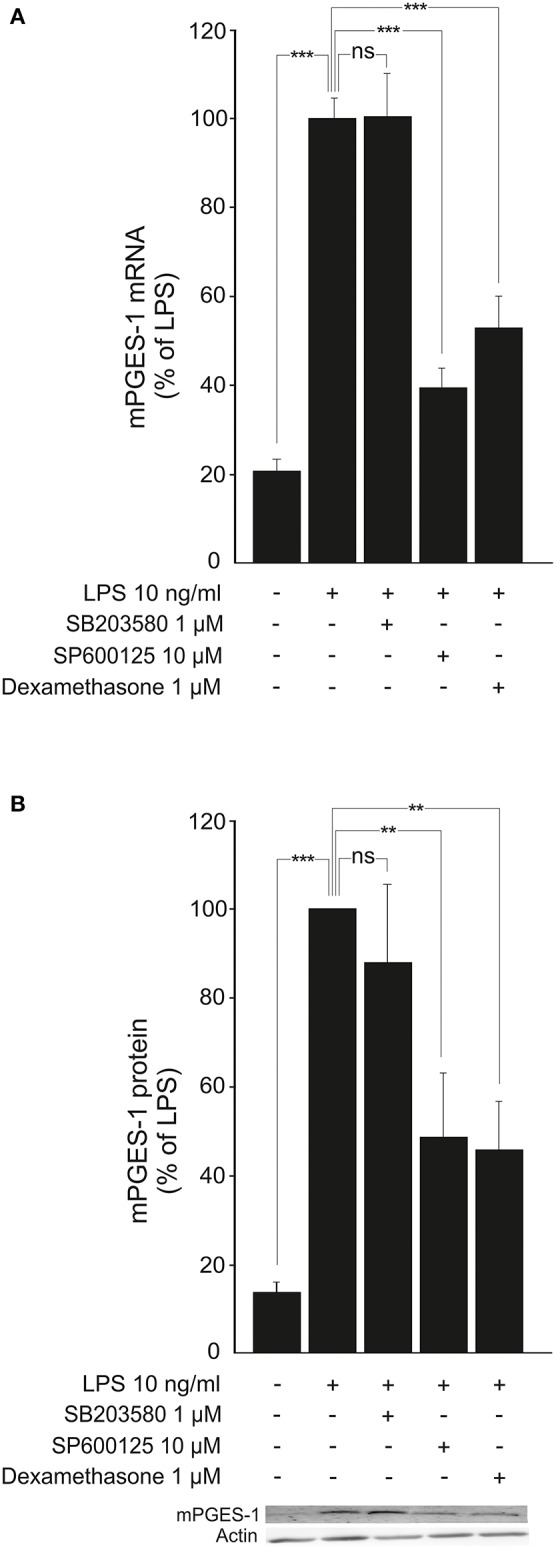
JNK inhibitor SP600125 inhibits the expression of mPGES-1 in activated macrophages. **(A)** Effects of a JNK inhibitor (SP600125), a p38 inhibitor (SB203580) and dexamethasone on mPGES-1 mRNA production in J774 murine macrophages. Cells were incubated with LPS and the compounds under investigation for 24 h. mPGES-1 mRNA levels were measured by quantitative RT-PCR and normalized against GAPDH mRNA levels. Results are expressed in arbitrary units, mPGES-1 mRNA levels in LPS-stimulated cells were set as 100% and the other values were related to that. Results are expressed as mean + SEM, *n* = 6–7. One-way ANOVA with Bonferroni's post-test was performed and statistical significance is indicated as ^***^P < 0.001 and ns = not significant. **(B)** Effects of a JNK inhibitor (SP600125), a p38 inhibitor (SB203580) and dexamethasone on mPGES-1 protein expression in J774 murine macrophages. Cells were stimulated with LPS and the compounds under investigation for 24 h. mPGES-1 protein levels were measured by Western blot analysis and actin was used as a loading control. Results are expressed in arbitrary units, mPGES-1 protein levels in LPS-stimulated cells were set as 100% and the other values were related to that. Results are expressed as mean + SEM, *n* = 5–6. One-way ANOVA with Bonferroni's post-test was performed and statistical significance is indicated as ^***^P < 0.001, ^**^P < 0.01 and ns = not significant. Shown is a representative gel of five with similar results.

Furthermore, the JNK inhibitor SP600125 (Bennett et al., 2001; Nieminen et al., 2006) but not the p38 inhibitor SB203580 (Cuenda et al., 1995; Shi et al., 2015) reduced LPS-induced mPGES-1 expression in a manner comparable to that of dexamethasone (Figures 6A,B).

Together, these data suggest that dexamethasone reduces mPGES-1 expression in classically activated macrophages in a manner dependent on enhanced MKP-1 expression and subsequently reduced JNK phosphorylation.

## Discussion

Microsomal prostaglandin E synthase-1 is an inducible inflammatory enzyme, the expression of which has been reported to be inhibited by glucocorticoids (Stichtenoth et al., 2001; Tuure et al., 2015). The present study extends the previous data by showing that the glucocorticoid effect on mPGES-1 is mediated through enhanced expression of the regulatory phosphatase MKP-1 and subsequent dephosphorylation of the MAP kinase JNK in inflammatory conditions.

MKP-1 deficient mice have a normal phenotype in resting conditions but they develop enhanced responses in inflammatory states. For example, they have impaired tolerance against bacterial endotoxin and when exposed to LPS, significantly higher levels of cytokines and other inflammatory factors are released and the inflammatory response is much more severe as compared to wild-type controls (Chi et al., 2006; Hammer et al., 2006; Zhao et al., 2006; Korhonen et al., 2011; Turpeinen et al., 2011). Those findings support a significant role for MKP-1 as an endogenous factor regulating and limiting excessive inflammatory responses and as a potential target to be increased with anti-inflammatory treatments. In the present experiments with cells from wild-type and MKP-1 deficient mice, we found that MKP-1 mediates the suppression by dexamethasone of mPGES-1 expression in activated macrophages. This effect was also translated to *in vivo* conditions, as dexamethasone reduced mPGES-1 expression in inflamed paw tissue in wild-type but not in MKP-1 deficient mice. Dexamethasone also suppressed inflammatory edema in wild-type but not in MKP-1 deficient mice. Although this is dependent on several inflammatory factors (Naidu et al., 2010; Zar et al., 2014; Chen et al., 2016), the reduced mPGES-1 expression may contribute to the anti-inflammatory effect of dexamethasone because mPGES-1 inhibitors have been reported to attenuate inflammatory paw edema in experimental models (Koeberle et al., 2009; Siemoneit et al., 2011).

In addition to its suppressive effect on mPGES-1, dexamethasone augmented the expression of MKP-1 when introduced to wild-type cells in the absence or in the presence of LPS, as shown also earlier (Abraham et al., 2006; Shipp et al., 2010; Zhu et al., 2010; Prabhala et al., 2016; Keränen et al., 2017). MKP-1 is an early response gene, the expression of which is transiently increased following exposure to inflammatory and cellular stress factors (Owens and Keyse, 2007; Boutros et al., 2008; Caunt and Keyse, 2013). In addition, MKP-1 promoter contains glucocorticoid responsive elements (Shipp et al., 2010) indicating that glucocorticoids may directly enhance MKP-1 transcription. MKP-1 expression is also regulated by various post-transcriptional mechanisms (Wong et al., 2005; Kuwano et al., 2008; Korhonen and Moilanen, 2014). As glucocorticoids have been shown not only to enhance but also to prolong MKP-1 expression (Keränen et al., 2017), they may regulate MKP-1 gene expression at post-transcriptional level in addition to their direct transcriptional effect.

MKP-1 regulates the inflammatory responses by inactivating MAP kinases p38 and JNK through dephosphorylation (Franklin and Kraft, 1997; Franklin et al., 1998; Chi et al., 2006; Zhao et al., 2006; Turpeinen et al., 2010, 2011; Comalada et al., 2012). Therefore, it is interesting that dexamethasone was found to reduce p38 and JNK phosphorylation in wild-type but not in MKP-1 deficient macrophages exposed to inflammatory stimulus. In support of our data, Abraham and co-workers reported that dexamethasone inhibits p38 and JNK phosphorylation in murine bone-marrow derived macrophages (Abraham et al., 2006), whereas in another study dexamethasone was found to downregulate p38 but not JNK or ERK phosphorylation (Bhattacharyya et al., 2007). These findings show that the glucocorticoid-induced enhancement in MKP-1 expression has functional consequences at the level of reduced MAP kinase phosphorylation, which is likely to result in deactivation of these inflammatory pathways possibly by a cell-type dependent manner.

The present findings provide support for MAP kinase JNK being a seminal downstream factor regulating the expression of mPGES-1 under inflammatory conditions in macrophages: dexamethasone was found to reduce the phosphorylation of both p38 and JNK kinases along with its stimulatory effect on MKP-1 expression. However, the JNK inhibitor but not p38 inhibitor attenuated the expression of mPGES-1. That is supported by the findings in human gingival fibroblasts (Yucel-Lindberg et al., 2006; Båge et al., 2010), in rat neonatal cardiomyocytes (Degousee et al., 2006) and in murine microglial cells (de Oliveira et al., 2008; He et al., 2016) in which JNK was found to be involved in the regulation of mPGES-1 expression. The effect may be cell type dependent because in human osteoarthritic chondrocytes stimulated with IL-1β, MAP kinases p38 and ERK had a crucial role in the regulation of mPGES-1 expression, whereas JNK was insignificant (Masuko-Hongo et al., 2004).

In inflammation, various pro-inflammatory cytokines and bacterial products are known to enhance mPGES-1 expression. The transcriptional mechanisms are not known in detail, but early growth response protein 1 (EGR-1) and nuclear factor kappa B (NF-kB) have been identified as key transcription factors for mPGES-1 (Koeberle and Werz, 2015). Activator protein 1 (AP-1) is one of the additional factors reported to be involved in the transcriptional activation of mPGES-1 (Moon et al., 2005; Jungel et al., 2007). This observation is relevant in the light of the present results because AP-1 is activated by JNK. Expression of mPGES-1 may also be regulated by JNK at post-transcriptional level as a JNK inhibitor has been found to destabilize mPGES-1 mRNA and reduce mPGES-1 protein levels in murine neonatal cardiomyocytes (Degousee et al., 2006). However, additional studies are needed to clarify the detailed mechanisms of how JNK regulates mPGES-1 expression.

In conclusion, dexamethasone was found to down-regulate mPGES-1 expression in classically activated macrophages through increased expression of the regulatory phosphatase MKP-1 and subsequently decreased phosphorylation of the MAP kinase JNK. These results extend the previous understanding of the molecular mechanisms regulating mPGES-1 expression in inflammatory conditions. The findings also highlight the potential of MKP-1 as an anti-inflammatory drug target.

## Author contributions

LT was involved in the conception and design of the study, laboratory and statistical analyses, analysis and interpretation of the data and drafted the manuscript. MH contributed to the conception and design of the study, laboratory analyses, animal experiments, analysis and interpretation of the data and in the writing of the manuscript. BW contributed to the analysis and interpretation of the data and in the writing of the manuscript. EM supervised the study and contributed to the conception and design of the study, in the analysis and interpretation of the data and in the writing of the manuscript. All authors approved the final version of the manuscript.

### Conflict of interest statement

The authors declare that the research was conducted in the absence of any commercial or financial relationships that could be construed as a potential conflict of interest.
